# Synergistic interaction network between the snR30 RNP, Utp23, and ribosomal RNA during ribosome synthesis

**DOI:** 10.1080/15476286.2022.2078092

**Published:** 2022-06-01

**Authors:** Timothy J. Vos, Ute Kothe

**Affiliations:** a Department of Chemistry, University of Manitoba, Winnipeg, Manitoba, Canada; b Alberta RNA Research and Training Institute (ARRTI), Department of Chemistry & Biochemistry, University of Lethbridge, Lethbridge, Alberta, Canada

**Keywords:** Ribosome biogenesis, RNA binding, Cbf5, RNA–protein interaction, H/ACA RNA, snoRNA

## Abstract

snR30/U17 is a highly conserved H/ACA RNA that is required for maturation of the small ribosomal subunit in eukaryotes. By base-pairing to the expansion segment 6 (ES6) of 18S ribosomal RNA (rRNA), the snR30 H/ACA Ribonucleoprotein (RNP) indirectly facilitates processing of the precursor rRNA (pre-rRNA) together with other proteins such as Utp23 and other RNAs acting as ribosome assembly factors. However, the details of the molecular interaction network of snR30 and its binding partners and how these interactions contribute to pre-rRNA processing remains unknown. Here, we report the *in vitro* reconstitution of a *Saccharomyces cerevisiae* snR30 RNP and quantitative characterization of the interactions of snR30, H/ACA proteins, the Utp23 protein and ES6 of the 18S rRNA. The snR30 RNA is bound tightly by both H/ACA proteins and Utp23. We dissected the importance of different 18S rRNA regions for snR30 RNP binding and demonstrated that the snR30 complex is tightly anchored on the pre-rRNA through base-pairing to ES6 whereas other reported rRNA binding sites do not contribute to the affinity of the snR30 RNP. On its own, the ribosome assembly factor Utp23 binds in a tight, but unspecific manner to RNA. However, in complex with the snR30 RNP, Utp23 increases the affinity of the RNP for rRNA revealing synergies between snR30 RNP and Utp23 which are enhancing specificity and affinity for rRNA, respectively. Together, these findings provide mechanistic insights how the snR30 RNP and Utp23 cooperate to interact tightly and specifically with rRNA during the early stages of ribosome biogenesis.

## Introduction

Ribosome biogenesis is a crucial process responsible for creating the large ribonucleoprotein (RNP) complex that is synthesizing all proteins. The process of ribosome biogenesis is highly conserved in eukaryotes such as *S. cerevisiae* and humans [1]. After beginning in the nucleolus and continuing in the nucleoplasm, ribosome synthesis ends in the cytoplasm and requires hundreds of temporary factors to come together and depart at timed intervals to form the two mature ribosome subunits [2]. The 35S rRNA precursor (47S in humans) is transcribed by RNA polymerase I [3] and contains three of the four rRNA fragments (the 18S, 5.8S, and 25S (28S in humans)), two internal transcribed spacers (ITS1 and ITS2), and two external transcribed spacers (5ʹ ETS and 3ʹ ETS).

Ribosome formation begins co-transcriptionally with the formation of the 90S pre-ribosome on the 5ʹ ETS [4–6]. As transcription starts, the sub-complex UTP-A binds the 5ʹ ETS and recruits a myriad of other complexes including but not limited to UTP-B, UTP-C, U3 small nucleolar RNP (snoRNP), and Mpp10 [7]. These complexes provide a temporary scaffold upon which the final ribosomal subunit can mature. In conjunction with this fast and early process, H/ACA and C/D snoRNPs act as modification complexes and introduce numerous pseudouridines and 2ʹ-O-methylations into the rRNA [8,9]. In yeast, four snoRNPs play a special role beyond rRNA modification: The C/D box U3, U14, snR10, and snR30 (U17 in humans). All of these snoRNPs except snR10 produce a lethal growth phenotype when depleted from the cell [10–12]. This phenotype arises from defects in processing pre-rRNA at sites A0, A1, and A2. The role of U3 snoRNA is understood best as it binds to the central pseudoknot of the 18S rRNA and prevents premature and/or mis-folding [13,14].

**Figure 1.**
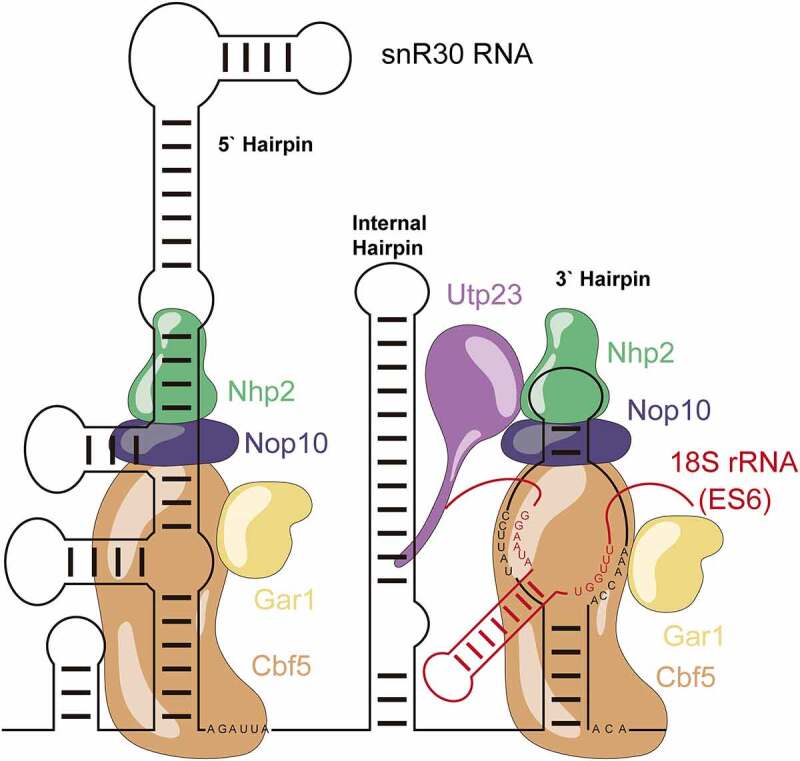
**Schematic structure of the snR30 RNP**. This representation displays the secondary structure of snR30 comprised of the 5ʹ, internal and 3ʹ hairpins as well as the associated H/ACA proteins (Cbf5 – orange, Nop10 - blue, Gar1 – yellow, and Nhp2 - green). The snR30 RNP also interacts with the ribosome assembly factor Utp23 (purple). The m1 and m2 motifs in the 3ʹ-hairpin base-pair with expansion segment 6 (ES6) of 18S rRNA (rm1 and rm2 motifs, red). Nucleotide sequences for the H and ACA boxes as well as the m1, m2 (snR30) and rm1, and rm2 (18S rRNA) sequences are displayed.

The other special snoRNPs (U14, snR10, snR30) have been proposed to form an interaction network utilizing the eukaryotic expansion segments [15]. Of them, snR30 is of special note due to its unique mode of interaction with the ribosomal RNA. Instead of introducing a pseudouridine like other H/ACA snoRNPs by binding the pre-rRNA across the distal half of the pseudouridylation pocket, snR30 binds in the inverse orientation preventing it from introducing a pseudouridine into the pre-rRNA (Fig. 1) [16]. The snR30 RNA contains highly conserved motifs named m1 and m2 (537–545 & 589–595) which bind to the corresponding rm1 and rm2 motifs (801–806 & 836–841) in the eukaryotic expansion segment 6 (ES6) of the central domain of the 18S rRNA [16].

snR30 has multiple features that distinguish it from standard H/ACA snoRNAs [17]. Instead of two hairpins, snR30 has an internal hairpin, a leader sequence, and an extended 5ʹ hairpin which contains an unpaired bulge similar to the so-called pseudouridylation pocket in standard H/ACA snoRNAs [17,18]. Notably, the 5ʹ or internal hairpins of snR30 can be deleted [16]. When snR30 binds to the pre-rRNA, it forms a new hairpin in the rRNA between the rm1 and rm2 motifs that is not seen in the mature fold of the 18S rRNA [19]. With its extended structure, snR30 can make two more interactions with the 5ʹ and internal hairpins. snR30ʹs 5ʹ hairpin binds to ES6H1 of the 18S rRNA (called C2 interaction site) while the internal hairpin interacts with helices 25 and 26 of the 18S rRNA (C3 site) [15]. It remains unknown what the function of these secondary binding sites is and whether they contribute to stabilization of the snR30 RNP or chaperoning the rRNA during ribosome biogenesis. Furthermore, snR30ʹs interaction with pre-rRNA is essential for the recruitment of the proteins Utp23 and Kri1 to the pre-ribosome [20].

Utp23 is an essential ribosome biogenesis factor with a degenerate PIN domain in yeast that cannot cleave RNA [21]. However, it does contain an essential CCHC-Zinc binding finger, an unstructured C-terminal tail, and a highly basic N-terminal alpha helix. The three cysteine residues of the zinc-finger are essential for Utp23 function and the C-terminal tail binds to snR30 *in vivo* through a conversed motif (PNPLSVKKKK) [21]. The binding of Utp23 to snR30 occurs primarily within the internal hairpin of snR30 with additional interaction with the 3ʹ hairpin [19]. Utp23ʹs conserved C-terminal tail motif is essential for binding the snR30 RNP and its subsequent recruitment to the pre-ribosome [21]. On the pre-ribosome, Utp23 contacts exclusively the ES6, primarily ES6 hairpin 3 (ES6H3) and H22 [19]. Furthermore, Utp23 can both bind to itself forming a homodimer as well as its paralog Utp24 (Fcf1p) [19]. Unlike Utp23, Utp24 has a fully functional PIN domain that is essential for ribosome biogenesis as Utp24 can cleave the coupled sites A1 and A2 during ribosome biogenesis.

Through forming an extensive protein–RNA interaction network, snR30 appears to be crucial in the folding and maturation of the ES6, as well as the eventual maturation of the small subunit, but the molecular details of snR30ʹs interaction network are only poorly understood. Utilizing the previously published *in vitro* reconstitution of a yeast H/ACA snoRNP complex, we are herein characterizing the interaction of snR30 with the 18S pre-rRNA. Further experiments with Utp23 reveal that both Cbf5-Nop10-Gar1-Nhp2 and Utp23 have a strong, unspecific affinity to RNA. Additionally, the snR30 RNP is able to bind specifically to the rm1/rm2 motif in the ES6 but not to other regions of the rRNA. This interaction is further stabilized by Utp23.

## Materials and methods

### Materials

[C5-^3^H]-UTP was purchased from Moravek Biochemicals. All chromatography materials are from GE Healthcare. Oligonucleotides were ordered from Integrated DNA Technologies (IDT) and synthesized plasmids were ordered from Genewiz. All other chemicals and enzymes were purchased from Fisher Scientific.

### Molecular cloning

The genes for CBF5, NOP10, NHP2, and GAR1 were cloned as previously described [22]. The sequence encoding UTP23 was codon-optimized for *E. coli*, synthesized and cloned into pGEX-5x-3 by Genewiz. The UTP23 gene is inserted between the BamHI and EcoRI sites such that a GST-tag is fused unto the N-terminus. Following synthesis, the pGEX-5x-3-UTP23 plasmid was transformed into *E. coli* BL21 (DE3) cells (NEB).

The genes for SNR30 and RDN37 were amplified from *S. cerevisiae* genomic DNA and cloned into pUC19 vectors restricted with SmaI using the following primers: snR30F: 5ʹ-GCTAATACGACTCACTATAGGGAACCATAGTCT CGTGCTAGTTCGGTACTATACAGGG-3ʹ; snR30R: 5ʹ-mAmGATGTCTGCAGTATGGTTTTACCCAAATGATCATGGACC-3ʹ and RDN37F: 5ʹ-ATGCGAAAGCAGTTGAAGACAA GTTCG-3ʹ; RDN37R: 5ʹ-CAAATCCTTTCACGCTCGGGAAGC-3ʹ.

To remove the internal hairpin of snR30, deletion mutagenesis was performed on pUC19-SNR30 using the primer set 5ʹ-phosCCGCAGTATATTCCTAAACACTAT-3ʹ and 5ʹ- CTTAATCTAAGTTAAACTCGTCAACG −3ʹ. The PCR product was isolated by Dpn1 digestion followed by ligation and transformation into DH5α *E. coli* (NEB). After miniprepping, the new plasmid pUC19-SNR30ΔIH was created.

### Protein expression and purification

The protein complex of Cbf5, Gar1, Nop10, and Nhp2 was purified as previously described [22]. In short, Cbf5 and Nop10 were co-expressed in *E. coli* BL21 (DE3) cells (NEB). Before cell opening and protein purification, BL21 DE3 cells expressing Gar1 were mixed with the Cbf5-Nop10-expressing cells in a 1:1 ratio and underwent two-step affinity purification using glutathione- and nickel-Sepharose.

The Utp23 purification was adapted from a previously published method [23]. *E. coli* BL21 (DE3) cells containing pGEX-5x-3-UTP23 were grown at 37°C until an OD_600_ of 0.6–0.8. Expression was induced by addition of isopropyl β-D-1-thiogalactopyranoside (IPTG) to a final concentration of 1 mM, and cell growth was continued at 18°C overnight. The next morning, cells were collected by centrifugation at 5,000 RCF.

The cell pellet was resuspended in cell opening buffer (20 mM Tris (pH 7.6), 150 mM NaCl, 5 mM MgCl_2_, 10% glycerol, and 0.1% NP-40) supplemented with 1 mM DTT and 1 mM PMSF. Lysozyme was added to the cell mixture which was allowed to incubate on ice for 30 minutes. Sodium deoxycholate was added, and the cells were lysed by sonication (Branson Sonifier 450) with five intervals at duty cycle 60% for two minutes each. Cell lysate was clarified by centrifugation at 30,000 RCF for 20 minutes. The clarified lysate was transferred to Glutathione Sepharose Fast Flow resin. The resin was washed with 20 column volumes (CV) of cell opening buffer. To remove chaperone contamination, the resin was washed with 1 CV of opening buffer supplemented with 5 µM ATP and 0.2 mg/ml denatured BSA which was previously boiled at 95°C and snap cooled on ice. The resin was washed again with 5 CV of opening buffer followed by another 5 CV of opening buffer supplemented with an extra 850 mM NaCl (total 1 M NaCl). Finally, the resin was washed once again with 15 CV of wash buffer (cell opening buffer with 0.1 mM DTT and substituting 0.1% Tween 20 for NP40). To elute the protein, 20 mM reduced glutathione was added to the wash buffer.

Utp23 elutions were concentrated using Vivaspin ultrafiltration devices (10kDa molecular weight cut-off). The concentrated protein was further purified by size exclusion chromatography using a Superdex 75 XK 26/100 column in wash buffer. The fractions containing Utp23 were collected, combined, and concentrated again. The purity and concentration of the protein were determined by sodium-dodecyl sulphate polyacrylamide electrophoresis (SDS-PAGE) and Bradford assay respectively.

### In vitro transcription and purification of RNA

The genes encoded in plasmids pUC19-SNR30, pUC19-SNR30ΔIH, and pUC19-RDN37 were amplified to create the templates used for *in vitro* transcription. The plasmid, product, and primers used are summarized in Table 1. All products marked with an asterisk (Table 1) were further amplified using the T7 promoter F oligo (5ʹ-GCTAATACGACTCACTATAGGG-3ʹ) and the same reverse primer as before.

**Table 1.** Summary of plasmids, primers and DNA products for *in vitro* transcription

Plasmid	DNA Product	Primer names	Primer sequences 5ʹ-3ʹ
pUC19-SNR30	snR30 full-length	snR30 F snR30 R	GCTAATACGACTCACTATAGGGAACCATAGTCTCGTGCTAGTTCGGTACTATACAGGG mAmGATGTCTGCAGTATGGTTTTACCCAAATGATCATGGACC
pUC19-SNR30	snR30 Δ5ʹ	snR30Δ5ʹ snR30 R	GCTAATACGACTCACTATAGGTAGGACGCATGATCTTGAGCTCTTTTCCTATACTTTG mAmGATGTCTGCAGTATGGTTTTACCCAAATGATCATGGACC
pUC19-SNR30	snR30 Δ5ʹΔIH	snR30Δ5ʹΔIH F snR30 R	GCTAATACGACTCACTATAGGGCAGTATATTCCTAAACACTATGAAAT mAmGATGTCTGCAGTATGGTTTTACCCAAATGATCATGGACC
pUC19-SNR30ΔIH	snR30 ΔIH	snR30 F snR30 R	GCTAATACGACTCACTATAGGGAACCATAGTCTCGTGCTAGTTCGGTACTATACAGGG mAmGATGTCTGCAGTATGGTTTTACCCAAATGATCATGGACC
pUC19-RDN37	C2-H22/23*	C2 F H22/23 R	TAATACGACTCACTATAGGGATTTTTTCGTGTACTGGATTTCCAACGGG GAAAACGTCCTTGGCAAATGCTTTCG
pUC19-RDN37	C2-ES6H1*	C2 F ES6H1 R	TAATACGACTCACTATAGGGATTTTTTCGTGTACTGGATTTCCAACGGG TCCTGGTTCGCCAAGAGCC
pUC19-RDN37	ES6H1-ES6H3*	ES6H1 F ES6H3 R	TAATACGACTCACTATAGGGTTCTGGCTAACCTTGAGTCCTTG mTmCATTACGATGGTCCTAGAAACCAAC
pUC19-RDN37	ES6H2-H22/23*	ES6H2 F H22/23 R	TAATACGACTCACTATAGGGTTACTTTGAAAAAATTAGAGTGTTCAAAGCAGGCG GAAAACGTCCTTGGCAAATGCTTTCG
pUC19-RDN37	ES6H2-ES6H3*	ES6H2 F ES6H3 R	TAATACGACTCACTATAGGGTTACTTTGAAAAAATTAGAGTGTTCAAAGCAGGCG mTmCATTACGATGGTCCTAGAAACCAAC
pUC19-RDN37	rm1-rm2	Rm1 F Rm2 R	GCTAATACGACTCACTATAGGGCATGGAATAATAGAATAGGACGTTTGGTTC CCTAGAAACCAACAAAATAGAACCAAACGTCCTATTCTATTATTCC
pUC19-RDN37	H22/23*	H22/23 F H22/23 R	TAATACGACTCACTATAGGGTTAATAGGGACGGTCGGGGG GAAAACGTCCTTGGCAAATGCTTTCG
pUC19-RDN37	H25/26*	H25/26 F H25/26 R	TAATACGACTCACTATAGGGCCGACTAGGGATCGGGTGG ACCCAAAGACTTTGATTTCTCGTAAGGTGC

As described in Wright et al. [24], the DNA products were purified using EZ-10 spin columns (BioBasic) and then used as the template in *in vitro* transcription reactions. The various snR30 RNAs were purified using a Superdex 200 10/300 GL column in 1x TE buffer pH 8.0. Isopropanol precipitation, ethanol wash, and resuspension in water were used to further purify the RNA.

Radioactive RNAs were created by transcribing in the presence of [C5-^3^H]-UTP. The RNA was purified using Nucleobond Xtra Midi anion exchange gravity columns (Macherey and Nagel). In brief, the column was washed with 100 mM Tris-acetate (pH 6.3), 10 mM MgCl_2_, 15% ethanol, 300 mM KCl. Following washing, the RNA was eluted in the same buffer with 1150 mM KCl. The eluted RNA was subjected to isopropanol precipitation and was resuspended in water. The concentration and specific activity of the RNA was determined by A_260_ absorbance (https://www.fechem.uzh.ch/MT/links/ext.html) and scintillation counting, respectively.

### Nitrocellulose filtration assays

Nitrocellulose filter binding to quantify protein binding to snR30 was performed as described by Caton et al. [22]. In short, radiolabeled snR30 RNAs were unfolded by incubation at 65°C for five minutes before being allowed to refold by slow cooling at room temperature. In all experiments, the concentration of the snR30 was constant and below the lowest protein concentration (0.27–0.42 nM of snR30 or 1.25 nM for snR30Δ5ʹΔIH for titration with Cbf5-Nop10-Gar1-Nhp2; 2 nM of snR30 or 4 nM for snR30Δ5ʹΔIH for titration with Utp23). The folded, radiolabeled RNA was incubated with protein of increasing concentration (Utp23: 0–300 nM; Cbf5-Nop10-Gar1-Nhp2: 0–15 nM) in Reaction Buffer (20 mM HEPES-KOH (pH 7.4), 150 mM NaCl, 1.5 mM MgCl2, and 10% (v/v) glycerol). The binding reactions were incubated at 30°C for 10 minutes before filtration through a nitrocellulose membrane. Following washing the membrane with 1 mL ice-cold Reaction Buffer, the amount of RNA-protein binding was determined by scintillation counting as previous [24].

For snR30 Ribonucleoproteins (RNPs) binding to radiolabeled rRNA fragments, the snR30 RNP complex was formed as before [22]. In brief, the proteins Cbf5, Nop10, Gar1 and Nhp2 are added in a 2:1 ratio relative to snR30 full-length and snR30ΔIH, but in a 1:1 ratio relative to snR30Δ5ʹ and snR30Δ5ʹΔIH. Reactions were set-up containing 5 nM snR30 RNP and increasing concentrations of radiolabeled rRNA fragments (10–1000 nM).

All experiments were completed in triplicate, the data points were averaged, and the percent binding was plotted against the titrant concentration. To determine the dissociation constant, the percentage of RNA binding is plotted against the titrant concentration and was fit in GraphPad Prism using the equation:

$$Y=B_{max}\times \;\left[S\right]\;÷\;\left(K_{D}+\;\left[S\right]\right)$$

Where [S] is the concentration of the titrated species, *K*_D_ is the dissociation constant, and *B*_max_ is the amplitude of the hyperbolic curve.

## Results

### snR30 interacts tightly with H/ACA proteins forming an H/ACA snoRNP

In analogy to H/ACA snoRNAs directing pseudouridine formation, snR30 is also hypothesized to form a snoRNP by binding two copies of the core four proteins Cbf5 (dyskerin or Nap57 in humans), Gar1, Nop10, and Nhp2 (L7Ae in Archaea) [17]. Previously, our group has shown that the core H/ACA protein complex of Cbf5-Nop10-Gar1-Nhp2 forms a tight interaction with the modification H/ACA snoRNA snR34 [22], corroborating previous evidence that guide RNAs are not exchanged by the core H/ACA proteins [25]. To verify that snR30 also binds tightly to the H/ACA core proteins, we utilized our previously published experimental system to reconstitute *S. cerevisiae* H/ACA snoRNPs from highly purified components including *in vitro* transcribed snR30 [22].

**Figure 2.**
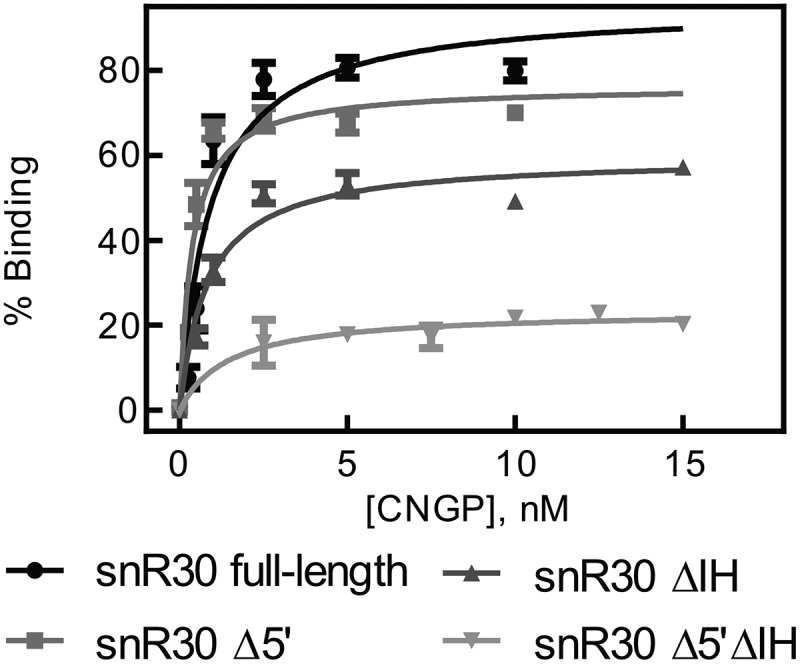
**Binding of snR30 to the H/ACA proteins Cbf5-Nop10-Gar1-Nhp2 (CNGP)**. Nitrocellulose filtration assays were conducted using snR30 full-length and truncations while titrating Cbf5-Nop10-Gar1-Nhp2 protein complex. Fitting to a hyperbolic equation yielded the dissociation constants and binding amplitudes (summarized in Table 2).

To quantify the interaction of snR30 with H/ACA core proteins, we utilized nitrocellulose filtration experiments, where the refolded, radioactively labelled snR30 RNA is incubated with the Cbf5-Nop10-Gar1-Nhp2 complex for 10 minutes at 30°C and subsequently filtered through a nitrocellulose membrane. Only snR30 that is bound to proteins is retained on the membrane during a washing step. The percentage of radiolabeled RNA bound to proteins on the membrane is determined by scintillation counting. Binding of snR30 to the proteins was measured in triplicate, and the percentage of protein-bound snR30 was averaged and plotted against the concentration of protein. To determine the dissociation constant (K_D_), the binding curve was fit with a hyperbolic function (equation 1, Materials and Methods). Thereby, we determined that the full-length snR30 interacts with a subnanomolar affinity with the H/ACA proteins (Fig. 2, Table 2). Similar to modification H/ACA snoRNPs, the tight binding of H/ACA proteins to snR30 is mediated by the trimeric Cbf5-Nop10-Gar1 complex as Nhp2 has a low affinity for snR30 (Fig. S1).

**Table 2.** Affinity of snR30 full-length and truncations binding to the H/ACA proteins Cbf5, Nop10, Gar1 and Nhp2. Dissociation constants (K_D_) were determined by nitrocellulose filtration (Fig. 2) and are reported with the standard deviation

snR30 Variant	Dissociation constant (nM)	Amplitude (% Binding)
snR30 (full-length)	0.9 ± 0.2	95 ± 6
snR30 Δ5ʹ	0.4 ± 0.1	76 ± 4
snR30 ΔIH	0.8 ± 0.1	60 ± 2
snR30 Δ5ʹΔIH	1.4 ± 0.8	23 ± 2

To test the role of the three hairpins in snR30 for protein interaction, three snR30 constructs were created lacking either the 5ʹ hairpin (snR30Δ5ʹ), internal hairpin (snR30ΔIH), or both (snR30 Δ5ʹΔIH). For all three snR30 variants, the affinity of the RNA to the four H/ACA core proteins remains unchanged (*K*_D_ = 0.4–1.4 nM; Fig. 2, Table 2) suggesting that the 3ʹ hairpin of snR30, which is common to all snR30 variants, binds the H/ACA proteins tightly. However, we noticed that the shortest snR30 variant, snR30 Δ5ʹΔIH, has a markedly reduced amplitude in binding proteins (23 ± 2%; Fig. 2) which could be a result of improper folding of this short snR30 variant. Notably, the 3ʹ hairpin is not the only H/ACA protein binding site in snR30 as we observed similar tight binding to an snR30 ∆3ʹ variant lacking the 3ʹ hairpin (Fig. S2). In conclusion, the H/ACA core proteins Cbf5-Nop10-Gar1-Nhp2 bind tightly to snR30, and the 3ʹ hairpin of snR30 is a major protein binding site confirming our hypothesis that snR30 interacts in the same manner with H/ACA proteins as modification H/ACA snoRNAs [22].

### Utp23 binds with a low nanomolar affinity to snR30

**Figure 3.**
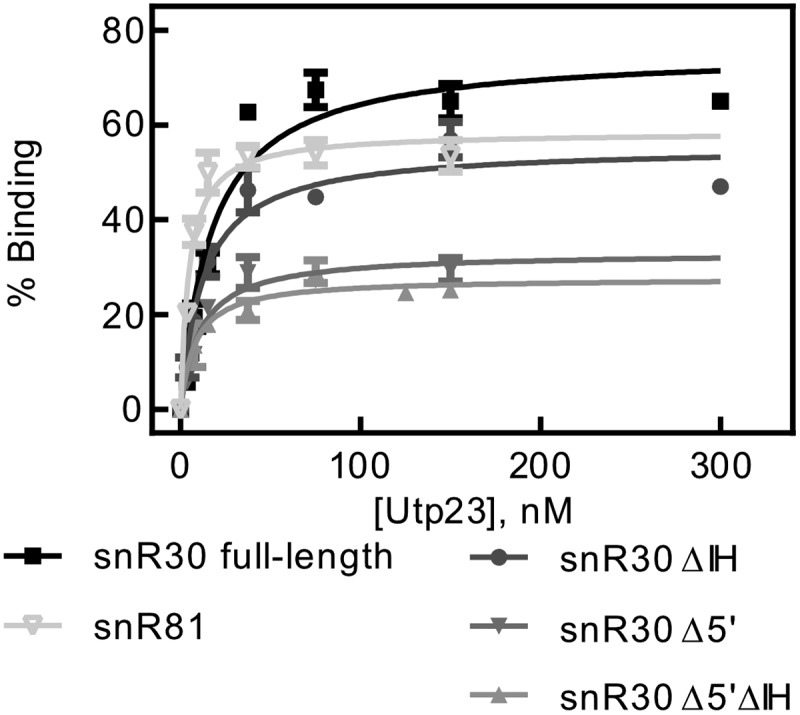
**Determining the affinity of Utp23 for snR30**. Utp23 was titrated against full-length snR30 and truncations thereof as well as the modification H/ACA snoRNA snR81 as control. Hyperbolic fitting provided the dissociation constants (see Table 3).

Utp23 binds to the snR30 RNP *in vivo* and makes extensive interactions with the internal hairpin [19–21], but the interaction of Utp23 with snR30 has not been quantitatively characterized so far. Therefore, we conducted nitrocellulose filtration assays to determine the affinity between Utp23 and snR30. As shown in Fig. 3, Utp23 binds tightly to full-length snR30 with an affinity of 17.5 ± 3.2 nM (Table 3) indicating that the interaction of snR30 with Utp23 is about 20-fold less tight than the interaction of snR30 with the core H/ACA proteins (Fig. 2, Table 2). To confirm that Utp23 binds to the internal hairpin of snR30 as reported, we then repeated the nitrocellulose filtrations with the different truncations of snR30. Interestingly, we observed almost no difference in Utp23 binding to snR30 full-length (*K*_D_ = 17.5 ± 3.2 nM) and snR30 ΔIH (*K*_D_ = 12.8 ± 2.4 nM) or the other variants of snR30 (Fig. 3, Table 3). This finding suggests that Utp23 can bind to the 3ʹ hairpin of snR30 (remaining in snR30 Δ5ʹΔIH) at least as well as the internal hairpin of snR30. Therefore, we asked next if Utp23ʹs high RNA affinity was specific to snR30 or whether it can bind other H/ACA snoRNAs in a similar manner. To test this, we utilized the modification H/ACA guide RNA snR81 in nitrocellulose filtration assays with Utp23. Notably, Utp23 interacts with snR81 as tightly (*K*_D_ = 4.7 ± 0.9 nM) as with snR30 supporting the hypothesis that the binding of Utp23 to snR30 in the absence of other factors may not be specific.

**Table 3.** Affinity of Utp23 for snR30 full-length and truncations. The dissociation constants for were measured by nitrocellulose filtration (Fig. 3) and are reported with the standard deviation

snR30 Variant	Dissociation constant (nM)	Amplitude (% Binding)
snR30 (full-length)	17.5 ± 3.2	76 ± 4
snR30 Δ5ʹ	11.0 ± 2.3	33 ± 2
snR30 ΔIH	12.8 ± 2.4	55 ± 3
snR30 Δ5ʹΔIH	8.1 ± 2.4	28 ± 1
snR81	4.7 ± 0.9	59 ± 2

### rRNA expansion segment 6 binds tightly to the snR30 RNP in vitro

snR30 binds two motifs, rm1 (801–806) and rm2 (836–841), on the 18S rRNA in a sequence-dependent fashion [26]. This region of the rRNA is in the eukaryotic-specific expansion segment ES6 which is flanked by helices 21 and 22/23 of the 18S rRNA. The region also contains the C2 site in H21 reported to be contacted by the snR30 RNP as well as potential other interactions with snR30 in H22 [15]. Notably, ES6 is bound by many different ribosome biogenesis factors including Utp24, the endonuclease responsible for the cleavages at A1 and A2 [27].

**Figure 4.**
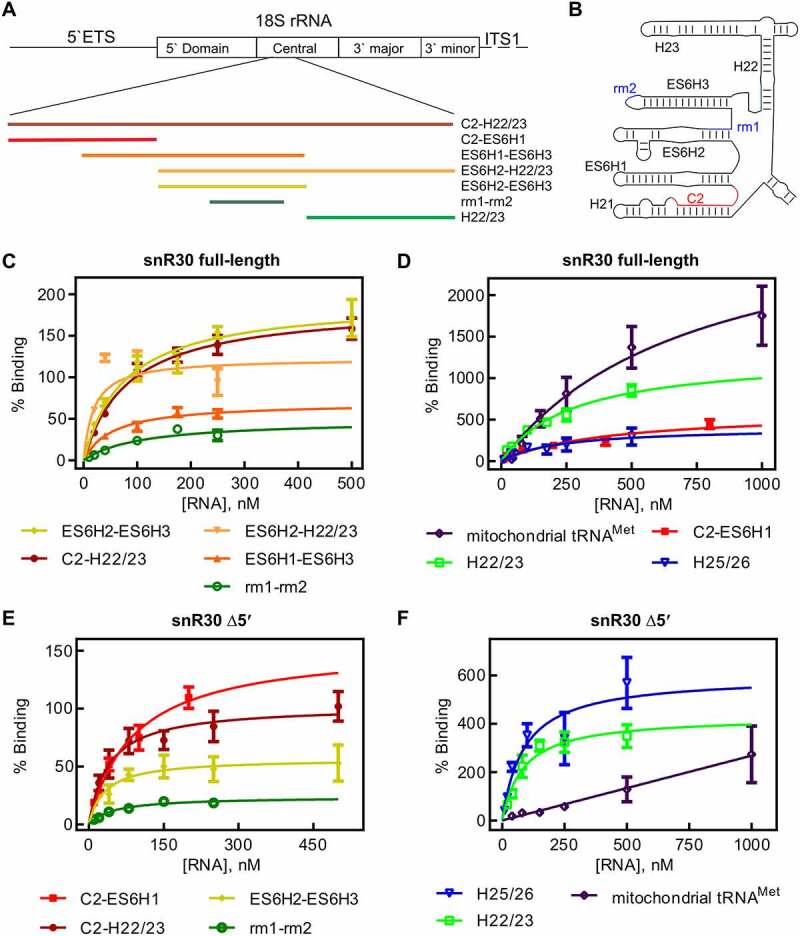
**18S rRNA fragments comprising and surrounding expansion segment 6 (ES6). A** Diagram of the 18S rRNA and surrounding regions indicating the various rRNA constructs used in this study. **B** Secondary structure of the ES6 and surrounding regions as observed in the mature 80S ribosome. **C** Binding of snR30 full-length complexed with Cbf5-Nop10-Gar1-Nhp2 to 18S rRNA fragments containing the rm1-rm2 motif as determined by nitrocellulose filtration. **D** Interaction of the full-length snR30 RNP with rRNA constructs lacking the rm1-rm2 motif as well as tRNA as control. **E** and **F** Similar nitrocellulose filtration assays using snR30 RNP reconstituted with snR30 Δ5ʹ. All dissociation constants were determined by hyperbolic fitting (smooth lines) and are summarized in Table 4.

To characterize the binding of the snR30 RNP to ES6 and the surrounding helices H21 and H22/23 of the 18S rRNA, varying rRNA fragments were constructed. Each ES6 construct includes one or more hairpins of the mature 18S rRNA fold (Fig. 4). The radiolabeled rRNA fragments were used in nitrocellulose filtration experiments upon incubation with snR30 full-length reconstituted with Cbf5-Nop10-Gar1-Nhp2 (snR30 RNP) to determine the affinity of the interaction.

The longest construct C2-H22/23 spanning nucleotides A661-C962 includes parts of H21 up to the entire H22 and contains both the rm1 and rm2, the C2 site and H22 which could also interact with snR30. Here, the radiolabeled rRNA is titrated against a constant concentration of snR30 full-length RNP. Fitting the data to the hyperbolic function, a low nanomolar dissociation constant was determined (84 ± 16 nM, Fig. 4, Table 4). To test whether the rRNA affinity of the snR30 RNP is dominated by the rm1 and rm2 motifs, a construct was created only containing the rm1-rm2 segment (C798-G846). The rm1-rm2 construct has an affinity of 106 ± 51 nM to the snR30 RNP which is not significantly lower than the affinity of the snR30 RNP for the C2-H22/23 construct indicating that the rm1 and rm2 motifs are anchoring the snR30 RNP on the 18S rRNA. Dissociation constants in this range agree with previously reported substrate RNA binding by the snR34 RNP (53–100 nM) [22].

**Table 4.** Affinity of the snR30 RNP complex binding to fragments of ES6 of 18S rRNA. The snRN30 RNP complex was titrated with rRNA fragments and binding was quantified by nitrocellulose filtration (Fig. 4). Dissociation constants are stated with the standard deviation

snR30 Variant	18S rRNA Fragment	Dissociation constant (nM)	Amplitude (% Binding)
snR30	C2-H22/23	84 ± 16	187 ± 11
	C2-ES6H1	405 ± 191	600 ± 140
	ES6H1-ES6H3	54 ± 19	70 ± 7
	ES6H2-ES6H3	77 ± 21	193 ± 16
	rm1-rm2	106 ± 51	48 ± 10
	ES6H2-H22/23	19 ± 10	123 ± 18
	H22/23	276 ± 72	1280 ± 170
	H25/26	225 ± 227	410 ± 190
	Mitochondrial tRNA^Met^	746 ± 405	3150 ± 950
snR30 Δ5ʹ	C2-H22/23	40 ± 15	102 ± 10
	C2-ES6H1	560 ± 700	480 ± 400
	ES6H1-ES6H3	ND	ND
	ES6H2-ES6H3	32 ± 18	57 ± 8
	rm1-rm2	50 ± 11	24 ± 2
	ES6H2-H22/23	ND	ND
	H22/23	84 ± 27	430 ± 46
	H25/26	92 ± 39	640 ± 93
	Mitochondrial tRNA^Met^	NC	NC

ND means the affinity was not measured. NC means the affinity could not be determined.

To further support the importance of rm1 and rm2 relative to the other ES6 rRNA hairpins, we generated additional ES6 constructs containing different hairpins of the ES6 surrounding the rm1 and rm2 motifs (ES6H1-ES6H3, U697-A859; ES6H2-ES6H3, U744-A859; ES6H2-H22/23, U744-C962, Fig. 4). These three 18S rRNA constructs all bind to the snR30 RNP with dissociation constants in the low nanomolar range (19–77 nM, Fig. 4, Table 4). To assess the binding of the snR30 RNP to rRNA constructs that do not possess the rm1 and rm2 constructs, additional portions of the ES6 were transcribed (C2-ES6H1, A661-A740; H22/23, U860-C962). These 18S rRNA fragments did not display the tight affinity of the rm1-rm2 containing constructs binding approximately 3-4-fold less tightly (405 ± 191 nM and 276 ± 72 nM for C2-ES6H1 and H22/23, respectively, Fig. 4, Table 4). Furthermore, the C2-ES6H1 and H22/23 constructs bound with extremely high amplitudes (600 ± 140% and 1280 ± 170%). This observation suggests that the rRNA fragments are interacting in an unspecific fashion with the snR30 RNP as many copies of the RNA bind to each RNP. As a control to determine the unspecific binding potential of the snR30 full-length RNP, mitochondrial tRNA^Met^ was used in nitrocellulose filtration experiments. The tRNA bound to the snR30 RNP with an affinity in the sub-micromolar range 750 ± 400 nM and an amplitude of 3150 ± 950% (Fig. 4, Table 4). Furthermore, we confirmed that addition of non-radiolabelled tRNA as competitor does not strongly change the observed affinity patterns (Fig. S3, Table S1). Therefore, we conclude that the snR30 full-length RNP can bind RNA in an unspecific fashion with a sub-micromolar affinity. Only rRNA fragments comprising the rm1-rm2 binding site have a high affinity for snR30 in low nanomolar range. Moreover, our results suggest that the secondary binding sites (C2, H22) do not contribute to the affinity of the snR30 RNP as much as the rm1-rm2 sites.

*In vivo*, the 5ʹ harpin of *S. cerevisiae* snR30 is dispensable for snR30ʹs function [16]. Hence, we tested if snR30Δ5ʹ would behave the same as snR30 full-length *in vitro* with respect to binding 18S rRNA. For the long rRNA construct C2-H22/23, we again observed a low nanomolar affinity of 40 ± 15 nM for binding to the snR30 RNP (Fig. 4, Table 4). Similar observations were made for ES6H2-ES6H3 and rm1-rm2 where the affinity remained in the low nanomolar range (32 ± 18 nM and 50 ± 11 nM, respectively). Unexpectedly, when the H22/23 rRNA fragment lacking the rm1/rm2 binding site was tested, it also bound the snR30Δ5ʹ RNP with high affinity (84 ± 27 nM; Fig. 4, Table 4). However, the H22/23 construct binds to snR30 RNP with a high amplitude (430 ± 46%) indicating the RNA is associating with the snR30Δ5ʹ RNP in an unspecific manner.

In summary, the snR30 RNP interacts tightly with the ES6 region of 18S rRNA and is anchored on the rRNA through base-pairing to the rm1/rm2 sites whereas the other predicted interaction sites (C2, H22) do not form high-affinity interactions with snR30 RNP on their own. Besides this specific, tight binding to rm1/rm2, we also discovered that the snR30 RNP can bind unspecifically and with high stoichiometries to other RNAs such as tRNA, but this interaction is characterized by a comparably low affinity in the high sub-micromolar range.

### Utp23 binds tightly to RNA stabilizing the snR30 RNP

**Figure 5.**
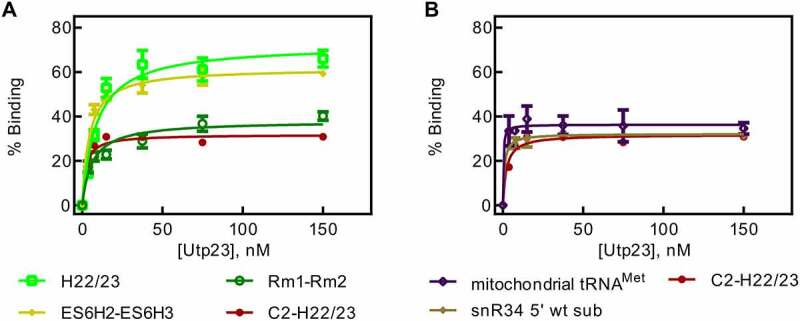
**Nitrocellulose filtration assays to quantify binding of Utp23 to 18S rRNA fragments. A** Utp23 binding to 18S rRNA fragments harbouring the rm1-rm2 motif. **B** Binding curves for Utp23 interaction with 18S rRNA which lacks the rm1-rm2 motif as well as two control RNAs.

Utp23 has been reported to bind to the 18S rRNA within ES6H3 and H22 [19]. Therefore, we sought to determine if Utp23 binds more tightly to these interaction sites than the surrounding areas of the 18S rRNA. Utilizing the same 18S rRNA fragments as for the snR30 RNP, we measured Utp23-rRNA binding using a low constant concentration of rRNA and titrating with Utp23 in nitrocellulose filtration experiments. For all rRNA constructs tested, Utp23 bound the RNA with uniform low affinity of 2.2 to 8.8 nM (Fig. 5, Table 5). This tight but non-specific binding was confirmed by measuring Utp23ʹs affinity to mitochondrial tRNA^Met^. Unexpectedly, Utp23 has an affinity below 2 nM for this tRNA (Fig. 5). However, adding non-radiolabelled tRNA as competitor does only slightly decrease the measured affinity of Utp23 for rRNA (Fig. S4). Next, we asked whether Utp23 binds preferably structured RNAs like the rRNA fragments and tRNA or whether it can also bind a short RNA that is predominantly single-stranded. Hence, we used a short 24-nt 25S rRNA fragment in nitrocellulose filtration that is the substrate of the snR34 H/ACA snoRNP catalysing pseudouridine formation [22]. Again, we observed a high affinity of less than 2 nM (Fig. 5, Table 5). In conclusion, Utp23 binds RNA very tightly in the low nanomolar range, but non-specifically.

**Table 5.** Affinity of Utp23 binding to 18S rRNA fragments. The dissociation constants and binding amplitudes were determined by nitrocellulose filtration (Fig. 5) and are summarized together with the standard deviation

18S rRNA Fragment	Dissociation constant (nM)	Amplitude (% Binding)
C2-H22/23	2.2 ± 0.8	32 ± 1
ES6H2-ES6H3	4.5 ± 0.7	62 ± 2
rm1-rm2	6.2 ± 1.6	38 ± 2
H22/23	8.8 ± 1.8	72 ± 4
mitochondrial tRNA^Met*^	<2 ± 1	36 ± 2
snR34 5ʹ wt sub*	<2 ± 1	32 ± 1

*Dissociation constant is lower than the RNA concentration in the reaction.

### rRNA binding by snR30 RNP in the presence of Utp23

**Figure 6.**
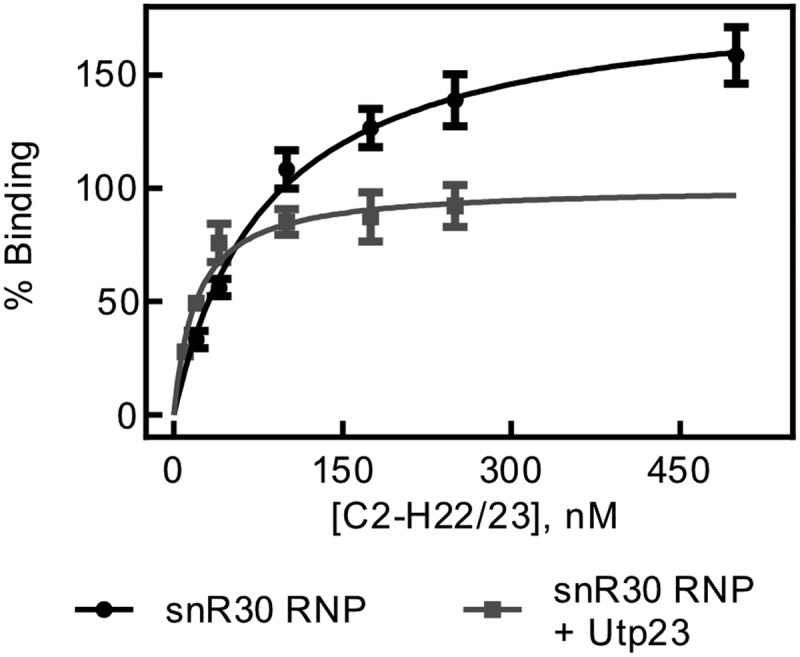
**rRNA binding by snR30 RNP in presence of Utp23**. Nitrocellulose filtration was used to quantify binding of the snR30 RNP (5 nM) to the region of 18S rRNA comprising the ES6 and flanking helices (C2-H22/23, Fig. 4)) in the presence of 5 nM Upt23. For comparison, binding of snR30 RNP to this rRNA fragment in the absence of Utp23 is also shown (same as in Fig. 4C). Hyperbolic fitting determined that snR30 RNP binds rRNA in the presence of Utp23 with a dissociation constant of 20 ± 5 nM and an amplitude of 101 ± 6%.

During ribosome biogenesis, many RNA and protein assembly factors cooperate and interact with each other such as the snR30 RNP and Utp23. As the snR30 RNP binds rm1-rm2 tightly and specifically, but Utp23 displays tight unspecific RNA binding, we asked whether Utp23 can further increase the affinity of the snR30 full-length complex to the C2-H22/23 rRNA fragment. To test this hypothesis, Utp23 was incubated with the snR30 full-length RNP in an equimolar ratio, and then titrated with C2-H22/23 rRNA in nitrocellulose filtration experiments. Interestingly, we determined a dissociation constant of 20 ± 5 nM and an amplitude of 101 ± 6% for the interaction of the complex of snR30 RNP and Utp23 with this rRNA fragment (Fig. 6. This experiment reveals that the binding of the snR30 full-length RNP to the ES6 rRNA construct is significantly tighter in the presence of Utp23 than in its absence (84 ± 16 nM). However, the observed affinity of snR30 RNP – Utp23 binding to the 18S rRNA is an order of magnitude lower than the binding of Utp23 alone to the same RNA (2.2 ± 0.8 nM). Similar trends were confirmed for binding of the snR30 RNP and Utp23 to the ES6H2-ES6H3 fragment of 18S rRNA both in the absence and presence of competitor tRNA (Fig. S5, Table S1). Therefore, there appears to be cooperation between the snR30 full-length RNP and Utp23 in binding to the rRNA.

## Discussion

Here, we report for the first time the *in vitro* reconstitution of the *S. cerevisiae* snR30 RNP from highly purified components and the quantitative characterization of crucial RNA-RNA and RNA-protein interactions of snR30. Using this highly defined experimental system allows us to dissect the functional importance of reported snR30 interaction representing a first critical step towards elucidating the molecular mechanism of snR30. Furthermore, we present the quantitative analysis of the ribosome biogenesis factor Utp23 interacting with snR30 and rRNA. Ultimately, this approach allowed us to begin reconstituting critical steps during ribosome formation by assessing the combined effect of the snR30 RNP and Utp23 on rRNA binding. In summary, this work represents a proof-of-concept for gaining mechanistic insight into the molecular interactions during ribosome biogenesis.

We show that the core H/ACA tetrameric proteins Cbf5-Gar1-Nop10-Nhp2 bind to snR30 with a sub-nanomolar affinity (Fig. 2, Table 2). This high affinity to snR30 is reminiscent of H/ACA proteins binding to other H/ACA guide RNAs to modify RNA suggesting that the snR30 RNP is stable once formed in the cell [22]. As Cbf5 recognizes the H and ACA boxes in H/ACA RNAs such as snR30, it is expected that two sets of the Cbf5-Gar1-Nop10-Nhp2 proteins can bind to full-length snR30 [22]. While nitrocellulose filtration experiments do not reveal the stoichiometry of protein binding to RNA, the truncations of snR30 clearly demonstrate that the Cbf5-Nop10-Gar1-Nhp2 protein complex binds with very high affinity to the 3ʹ hairpin of snR30 which harbours the m1 and m2 motifs and which precedes the ACA box. Moreover, deletion of the 3ʹ hairpin confirms that there is at least one additional binding site for H/ACA proteins in the 5ʹ or internal hairpin of snR30 (Fig. S2). Our ability to form an active, *in vitro* reconstituted snR30 RNP enables future studies further addressing its structure, function, interactions, and molecular mechanism during ribosome formation.

In addition, we demonstrate that highly purified Utp23 binds in a tight, yet non-specific fashion to RNA (Fig. 3, Table 3). It is surprising that Utp23 appears to bind RNA in an unspecific manner which could be mediated by the N-terminal positively charged alpha helix of Utp23 [21]. This observation raises the question how Utp23 can find its specific site of action during ribosome biogenesis in the nucleolus without staying bound to incorrect areas of rRNA and snoRNAs. Importantly, Utp23 does not act in isolation in the cellular environment but interacts with other proteins such as Nhp2 [19]. In the cell, Utp23 would not bind to isolated snR30 but to the entire snR30 RNP. Presumably, the protein interaction partners of Utp23 contribute to conferring some specificity to RNA binding by Utp23 by selectively targeting this protein to specific RNA regions. For example, in the snR30 RNP, the H/ACA proteins are expected to bind to the 5ʹ and 3ʹ hairpins as designated by the H and ACA boxes respectively, preventing other proteins from accessing snR30 in these regions. In contrast, the internal hairpin likely remains available for other interactions. Thus, Utp23 could be specifically positioned in the context of the snR30 RNP by interacting both with the available internal hairpin and Nhp2. In this context, the interaction of Utp23 and Nhp2 could be the most important interaction as the internal hairpin of snR30 is not required for snR30ʹs function in recruiting Utp23 to the pre-ribosome [16].

The high affinity of Utp23 for RNA was also unexpected as previously the affinity of Utp23 to snR30 was estimated to be in the low micromolar range based on electrophoretic mobility shift assays [19,21]. These different observations can possibly be explained by rapid association and rapid dissociation of Utp23 to RNA. Such a dynamic RNA binding mode can lead to high RNA affinity based on fast association. But the fast dissociation of Utp23 could allow Utp23 to search for correct RNA binding partners in the cell without staying bound to an incorrect RNA for too long time. During nitrocellulose filtration experiments, the membrane is washed only for a few seconds allowing us to detect RNA-protein complexes that dissociate rapidly. However, electrophoresis mobility shift assays are conducted on the timescale of hours such that the RNA-protein complex may dissociate during electrophoresis resulting in an underestimation of the affinity.

Regarding interactions with pre-rRNA, the reconstituted snR30 RNP allowed us to probe for the relative importance of interactions of this ribonucleoprotein with different regions of the rRNA during ribosome biogenesis (Fig. 4, Table 3). Notably, the snR30 RNP binds with low nanomolar affinity to rRNA fragments containing the rm1-rm2 motifs which base-pair with the m1-m2 motifs in snR30 confirming the importance of this interaction. In contrast, the C2 interaction site located in H21 of 18S rRNA alone does not bind with similar tight affinity to the snR30 RNP, and the same is true for H22/23 flanking ES6 of 18S rRNA. This quantitative comparison thus demonstrates that the base-pairing to the rm1-rm2 sites not only confirms specificity, but also contributes the most binding energy to the recruitment of the snR30 RNP to rRNA during ribosome biogenesis. The other reported interactions of snR30 with rRNA (C2 in H21 and C3 in H25/26) likely represent contacts that may orient the snR30 RNP relative to the pre-ribosome, but they are not energetically contributing to the tight binding of the snR30 RNP to rRNA [15]. Interestingly, we observed that the snR30 RNP also interacts with a low affinity and in an unspecific manner with RNA as evident by its interaction with tRNA and the high amplitudes in some binding experiments. These low-affinity unspecific interactions with other rRNA segments or other snoRNAs may further stabilize the binding of snR30 to the pre-ribosome in the cell in addition to its specific and tight interaction with the m1 and m2 sites in ES6.

Moreover, we determined the affinity of Utp23 to rRNA fragments and again observed an unexpected high affinity, yet non-specific RNA binding. This tight RNA binding by Utp23 was observed both for rRNA constructs containing its reported binding site in ES6H3 and H22 in 18S rRNA as well as other RNAs that did not contain this region (Fig. 5, Table 5) [19]. Again, this observation suggests that other factors assist Utp23 in locating its site of action during ribosome biogenesis . The RNA specificity of Utp23 could be increased by its interaction with other proteins acting as ribosome assembly factors such as Utp24 or Kri1 [20]. In addition, the specificity of Utp23 is very likely improved by the snR30 RNP. Indeed, we noticed that the complex of snR30 RNP and Utp23 binds to ES6 of 18S rRNA with an intermediate affinity compared to the snR30 RNP and Utp23 alone (Fig. 6). This finding demonstrates that Utp23 increases the affinity of the snR30 RNP to rRNA whereas the snR30 RNP contributes specificity for binding ES6 through base-pairing. Thereby, we reveal the importance of this interaction network between Utp23, snR30 RNP and rRNA where all partners synergistically contribute to tight and specific binding required for ribosome biogenesis. It is interesting to compare our finding that Utp23 further increases the affinity of the snR30 RNP to pre-rRNA with a previous report that Utp23 is required for the release of snR30 from pre-ribosomal particles [20]. Our quantitative analysis demonstrates that Utp23 does not directly dissociate snR30 from pre-rRNA. Instead, it is conceivable that Utp23 is required for an event that precedes dissociation of snR30 while the removal of snR30 from the pre-ribosome is likely catalysed by Rok1 [28].

In summary, we have characterized important protein-RNA and RNA-RNA interactions between Utp23, snR30 and rRNA that determine the specificity and affinity of recruiting ribosome assembly factors such as Utp23 and the snR30 RNP to the nascent pre-rRNA during ribosome biogenesis. Such quantitative biochemical studies can in the future be expanded to further explore the interactions and functions of the snR30 RNP, Utp23, and other related ribosome biogenesis factors during pre-rRNA folding and processing. In particular, it will be interesting to probe interactions of snR30 and Utp23 with Utp24 which is responsible for pre-rRNA processing at sites A1 and A2 [27]. Similarly, our experimental system can be utilized to characterize the yeast H/ACA snoRNA snR10 which is involved in both pre-rRNA modification and processing and which is critical for *S. cerevisiae* fitness at cold temperatures [29,30]. In conclusion, the reconstitution and characterization of the snR30 RNP presented here lays the foundation to address mechanistic question during the early stages of eukaryotic ribosome formation.

## Supplementary Material

Supplemental Material

## Data Availability

The authors confirm that the data supporting the findings of this study are available within the article and its supplementary materials.

## References

[1] Bassler J, Hurt E. Eukaryotic ribosome assembly. Annu Rev Biochem. 2019;88(1):281–306. 30566372 10.1146/annurev-biochem-013118-110817

[2] Woolford JL Jr., Baserga SJ. Ribosome biogenesis in the yeast *Saccharomyces cerevisiae*. Genetics. 2013;195(3):643–681. 24190922 10.1534/genetics.113.153197 PMC3813855

[3] Russell J, Zomerdijk JC. The RNA polymerase I transcription machinery. Biochem Soc Symp. 2006;73:203–216. DOI:10.1042/bss0730203 16626300 PMC3858827

[4] Perez-Fernandez J, Román A, De Las Rivas J, et al. The 90S preribosome is a multimodular structure that is assembled through a hierarchical mechanism. Mol Cell Biol. 2007;27(15):5414–5429. 17515605 10.1128/MCB.00380-07 PMC1952102

[5] Hunziker M, Barandun J, Petfalski E, et al. UtpA and UtpB chaperone nascent pre-ribosomal RNA and U3 snoRNA to initiate eukaryotic ribosome assembly. Nat Commun. 2016;7(1):12090. 27354316 10.1038/ncomms12090 PMC4931317

[6] Zhang LM, Wu C, Cai G, et al. Stepwise and dynamic assembly of the earliest precursors of small ribosomal subunits in yeast. Genes Dev. 2016;30(6):718–732. 26980190 10.1101/gad.274688.115 PMC4803056

[7] Chaker-Margot M, Barandun J, Hunziker M, et al. Architecture of the yeast small subunit processome. Science. 2017;355(6321):6321. 10.1126/science.aal1880 27980088

[8] Kiss-Laszlo Z, Henry Y, Bachellerie J-P, et al. Site-specific ribose methylation of preribosomal RNA: a novel function for small nucleolar RNAs. Cell. 1996;85(7):1077–1088. 8674114 10.1016/s0092-8674(00)81308-2

[9] Ganot P, Bortolin ML, Kiss T. Site-specific pseudouridine formation in preribosomal RNA is guided by small nucleolar RNAs. Cell. 1997;89(5):799–809. 9182768 10.1016/s0092-8674(00)80263-9

[10] Li HD, Zagorski J, Fournier MJ. Depletion of U14 small nuclear RNA (snR128) disrupts production of 18S rRNA in *Saccharomyces cerevisiae*. Mol Cell Biol. 1990;10(3):1145–1152. 2406561 10.1128/mcb.10.3.1145-1152.1990 PMC360983

[11] Hughes JM, Konings DA, Cesareni G. The yeast homologue of U3 snRNA. EMBO J. 1987;6(7):2145–2155. 3308452 10.1002/j.1460-2075.1987.tb02482.x PMC553607

[12] Bally M, Hughes J, Cesareni G. Snr30 - a new, essential small nuclear-Rna from Saccharomyces-Cerevisiae. Nucleic Acids Res. 1988;16(12):5291–5303. 2898766 10.1093/nar/16.12.5291 PMC336768

[13] Sharma K, Tollervey D. Base pairing between U3 small nucleolar RNA and the 5’ end of 18S rRNA is required for pre-rRNA processing. Mol Cell Biol. 1999;19(9):6012–6019. 10454548 10.1128/mcb.19.9.6012 PMC84488

[14] Dutca LM, Gallagher JE, Baserga SJ. The initial U3 snoRNA:pre-rRNA base pairing interaction required for pre-18S rRNA folding revealed by in vivo chemical probing. Nucleic Acids Res. 2011;39(12):5164–5180. 21349877 10.1093/nar/gkr044 PMC3130255

[15] Martin R, Hackert P, Ruprecht M, et al. A pre-ribosomal RNA interaction network involving snoRNAs and the Rok1 helicase. RNA. 2014;20(8):1173–1182. 24947498 10.1261/rna.044669.114 PMC4105744

[16] Atzorn V, Fragapane P, Kiss T. U17/snR30 is a ubiquitous snoRNA with two conserved sequence motifs essential for 18S rRNA production. Mol Cell Biol. 2004;24(4):1769–1778. 14749391 10.1128/MCB.24.4.1769-1778.2004 PMC344193

[17] Vos TJ, Kothe U. snR30/U17 small nucleolar ribonucleoprotein: a critical player during ribosome biogenesis. Cells. 2020;9(10):2195. 10.3390/cells9102195 PMC7601244 33003357

[18] Morrissey JP, Tollervey D. Yeast Snr30 is a small nucleolar Rna required for 18s ribosomal-Rna synthesis. Mol Cell Biol. 1993;13(4):2469–2477. 8455623 10.1128/mcb.13.4.2469-2477.1993 PMC359567

[19] Wells GR, Weichmann F, Sloan KE, et al. The ribosome biogenesis factor yUtp23/hUTP23 coordinates key interactions in the yeast and human pre-40S particle and hUTP23 contains an essential PIN domain. Nucleic Acids Res. 2017;45(8):4796–4809. 28082392 10.1093/nar/gkw1344 PMC5416842

[20] Hoareau-Aveilla C, Fayet-Lebaron E, Jády BE, et al. Utp23p is required for dissociation of snR30 small nucleolar RNP from preribosomal particles. Nucleic Acids Res. 2012;40(8):3641–3652. 22180534 10.1093/nar/gkr1213 PMC3333846

[21] Lu J, Sun MY, Ye KQ. Structural and functional analysis of Utp23, a yeast ribosome synthesis factor with degenerate PIN domain. RNA. 2013;19(12):1815–1824. 24152547 10.1261/rna.040808.113 PMC3860261

[22] Caton EA, Kelly EK, Kamalampeta R, et al. Efficient RNA pseudouridylation by eukaryotic H/ACA ribonucleoproteins requires high affinity binding and correct positioning of guide RNA. Nucleic Acids Res. 2018;46(2):905–916. 29177505 10.1093/nar/gkx1167 PMC5778458

[23] Schneider C, Anderson JT, Tollervey D. The exosome subunit Rrp44 plays a direct role in RNA substrate recognition. Mol Cell. 2007;27(2):324–331. 17643380 10.1016/j.molcel.2007.06.006 PMC7610968

[24] Wright JR, Keffer-Wilkes LC, Dobing SR, et al. Pre-steady-state kinetic analysis of the three *Escherichia coli* pseudouridine synthases TruB, TruA, and RluA reveals uniformly slow catalysis. RNA. 2011;17(12):2074–2084. 21998096 10.1261/rna.2905811 PMC3222121

[25] Wang C, Meier UT. Architecture and assembly of mammalian H/ACA small nucleolar and telomerase ribonucleoproteins. EMBO J. 2004;23(8):1857–1867. 15044956 10.1038/sj.emboj.7600181 PMC394235

[26] Fayet-Lebaron E, Atzorn V, Henry Y, et al. 18S rRNA processing requires base pairings of snR30 H/ACA snoRNA to eukaryote-specific 18S sequences. EMBO J. 2009;28(9):1260–1270. 19322192 10.1038/emboj.2009.79 PMC2664660

[27] Wells GR, Weichmann F, Colvin D, et al. The PIN domain endonuclease Utp24 cleaves pre-ribosomal RNA at two coupled sites in yeast and humans. Nucleic Acids Res. 2016;44(11):5399–5409. 27034467 10.1093/nar/gkw213 PMC4914098

[28] Bohnsack MT, Kos M, Tollervey D. Quantitative analysis of snoRNA association with pre-ribosomes and release of snR30 by Rok1 helicase. EMBO Rep. 2008;9(12):1230–1236. 18833290 10.1038/embor.2008.184 PMC2570499

[29] Tollervey D, Guthrie C. Deletion of a yeast small nuclear RNA gene impairs growth. EMBO J. 1985;4(13B):3873–3878. 3004976 10.1002/j.1460-2075.1985.tb04160.x PMC554743

[30] Tollervey D. A yeast small nuclear RNA is required for normal processing of pre-ribosomal RNA. EMBO J. 1987;6(13):4169–4175. 3327689 10.1002/j.1460-2075.1987.tb02763.x PMC553900

