# Supplementary material for: Synergistic interaction network between the snR30 RNP, Utp23, and ribosomal RNA during ribosome synthesis
Source: RNA Biol. 2022 Jun 1;19(1):764–73. doi: 10.1080/15476286.2022.2078092 (PMC9176245; doi:10.1080/15476286.2022.2078092)
Supplement: Supplemental Material [file KRNB_A_2078092_SM0098.pdf]

Supplemental Material

Synergistic interaction network between the snR30 RNP, Utp23, and ribosomal RNA during ribosome synthesis

Timothy J. Vos [1,2] and Ute Kothe [1,2*]

Timothy.vos@umanitoba.ca; ute.kothe@umanitoba.ca

Affiliation:

[1] Department of Chemistry, University of Manitoba, Winnipeg, Manitoba, R3T 2N2, Canada

[2] Alberta RNA Research and Training Institute (ARRTI), Department of Chemistry & Biochemistry, University of Lethbridge, Lethbridge, Alberta, T1K 3M4, Canada

Supplementary Results and Discussion

Interaction of snR30 with H/ACA proteins

We have previously shown that binding of the modification H/ACA snoRNA snR34 to the H/ACA proteins (Cbf5-Nop10-Gar1) is independent of the Nhp2 protein and that Nhp2 alone has a low affinity for snR34 [1]. Now, we asked whether the same interaction strengths are observed for binding of the H/ACA proteins to snR30 (Fig. S1). As hypothesized, the trimeric complex of Cbf5-Nop10-Gar1 binds snR30 with similar low nanomolar affinity as the complete set of all four H/ACA proteins including Nhp2 (Fig. 2 and S1A). When measuring the affinity of Nhp2 alone for snR30, we observe dissociation constants between 100 – 250 nM (Fig. S1B) which is in a similar order of magnitude as binding of Nhp2 to snR34[1]. In conclusion, snR30 displays similar properties as modification H/ACA snoRNAs with respect to binding H/ACA proteins since the interaction with Cbf5-Nop10-Gar1 is very tight and the interaction with Nhp2 is much weaker.

By deleting the 5` and internal hairpin of snR30, we have demonstrated that the H/ACA proteins can bind as expected to the 3` hairpin which harbors the pocket for base-pairing with rRNA (Fig. 2 and Table 2). In addition, we tested whether the 3` hairpin is the exclusive site in snR30 for H/ACA protein binding by deleting the 3` hairpin (snR30 Δ3`). Nitrocellulose filtration experiments revealed that H/ACA proteins continue to bind with low nanomolar affinity to snR30 when the 3` hairpin is deleted (Fig. S2). This result suggests that H/ACA proteins can also bind to the 5` or internal hairpin. In modification H/ACA snoRNAs, the Cbf5-Nop10-Gar1-Nop10 complex must bind to the 3` and the 5` hairpin which both harbor a pseudouridylation pocket and target different regions in rRNA for modification. Accordingly, it is expected that H/ACA proteins also bind to more than one hairpin in snR30, in particular since an H box and an ACA box sequence are located downstream of the 5` and 3` hairpins, respectively. *In vivo*, biogenesis of H/ACA snoRNPs follows a dedicated pathway where the H/ACA proteins are specifically located to the site of H/ACA snoRNA transcription [2]. Therefore, it is likely that the H/ACA proteins also bind snR30 co-transcriptionally at both the 5’ and 3’ hairpin including the adjacent H and ACA boxes *in vivo*.

rRNA binding in the presence of competitor RNA

Our systematic analysis of RNA binding to the snRN30 RNP and to Utp23 revealed specific binding of snR30 RNP to rRNA harboring the rm1 and rm2 sites and tight, but unspecific binding of Utp23 to RNA (Fig. 4 & 5, Table 4 & 5). At the same time, we noted weak unspecific binding of the snR30 RNP to tRNA suggesting that RNA binding to the snR30 RNP may be comprised of both a specific RNA binding and an unspecific RNA binding component. To better define the specificity of the snR30 RNP for rRNA with the rm1 and rm2 elements, we therefore repeated selected nitrocellulose filtration

experiments measuring binding of radiolabelled rRNA fragments to snR30 RNP in the presence of non-radiolabelled competitor tRNA (Fig. S3 and Table S1). As expected, the presence of the weakly binding tRNA influenced the binding curves slightly; whereas the K_D for C2-H22/23 remained unchanged, the affinity for ES6H2-ES6H3 decreased 4-fold (Table S1). Importantly, however, we observed the same trends as for the nitrocellulose filtration data in absence of tRNA: the rRNA constructs harboring the rm1-rm2 sites bind more strongly to the snR30 RNP than other rRNA fragments underlining the importance of the base-pairing interactions of snR30 and rRNA for anchoring the snR30 RNP on the pre-ribosome.

Similarly, we also assessed the affinity of Utp23 to rRNA in the presence of competitor tRNA. Again, we observed a slight increase in K_D as the tRNA can compete for binding to Utp23, but the affinity for C2-H22/23 remained in the low nanomolar range (9.0 nM, Table S1).

Lastly, we determined the affinity of the snR30 RNP in the presence of Utp23 for the ES6H2-ES6H3 rRNA fragment which contains the rm1 and rm2 sites (Figure S5, Table S1). Notably, we record an affinity of 31 ± 7 nM in the absence of competitor tRNA. Comparing this affinity to the binding of snR30 RNP alone to the ES6H2-ES6H3 rRNA fragment (K_D of 77 ± 21 nM, Table 4) again demonstrates that Utp23 increases the affinity of snR30 RNP for RNA. When we repeated the measurement in the presence of competitor tRNA, a dissociation constant of 58 ± 8 nM is observed which shows that rRNA binding to snR30 RNP and Utp23 is not strongly influenced by the competitor RNA.

Supplementary Materials & Methods

Preparation of snR30 Δ3`

snR30 lacking the 3` hairpin (snR30 **Δ**3`) was prepared by amplifying the coding region using the snR30 F primer (Table 1) and a specific snR30 **Δ**3` R primer (5`-mUmUTAATCTAAGTTAAACTCGTCAACGGGGCCACTTC-3`) followed by *in vitro* transcription in the presence of [C5-^{3}H]-UTP and purification as described.

Nitrocellulose filtration assays in the presence of competitor tRNA

To test for the specificity of rRNA binding, selected nitrocellulose filtration assays were conducted as described while adding 1 µM purified *E. coli* $tRNA^{Phe}$ (non-radioactive) as competitor RNA. $tRNA^{Phe}$ was prepared as previously described [3].

Supplementary References

1. Caton, E.A., et al., *Efficient RNA pseudouridylation by eukaryotic H/ACA ribonucleoproteins requires high affinity binding and correct positioning of guide RNA.* Nucleic Acids Res, 2018. **46**(2): p. 905-916.
2. Czekay, D.P. and U. Kothe, *H/ACA Small Ribonucleoproteins: Structural and Functional Comparison Between Archaea and Eukaryotes.* Front Microbiol, 2021. **12**: p. 654370.
3. Wright, J.R., et al., *Pre-steady-state kinetic analysis of the three Escherichia coli pseudouridine synthases TruB, TruA, and RluA reveals uniformly slow catalysis.* RNA, 2011. **17**(12): p. 2074-2084.

Table S1. Binding of rRNA fragments by snR30 RNP and Utp23 in the presence of competitor RNA. Nitrocellulose filtration assays were conducted in the presence of 1 μM tRNA (non-radioactive) as an unspecific competitor as shown in Figures S3-S5. Hyperbolic fitting yielded the dissociation constants (K_D) listed here with standard deviations. For comparison, the respective dissociation constants reported in the absence of competitor RNA are shown again (Table 4 & 5).

**Target RNA**	**Binding protein / complex**	**K_D, nM in presence of tRNA**	**K_D, nM in absence of tRNA**
C2-H22/23	snR30 wt RNP	88 ± 27	84 ± 16
H22/23	snR30 wt RNP	459 ± 160	276 ± 72
ES6H2-ES6H3	snR30 Δ5` RNP	133 ± 35	32 ± 18
rm1-rm2	snR30 Δ5` RNP	25 ± 8	50 ± 11
C2-H22/23	Utp23	9.0 ± 1.2	2.2 ± 0.8
ES6H2-ES6H3	snR30 wt RNP & Utp23	58 ± 8	31 ± 7

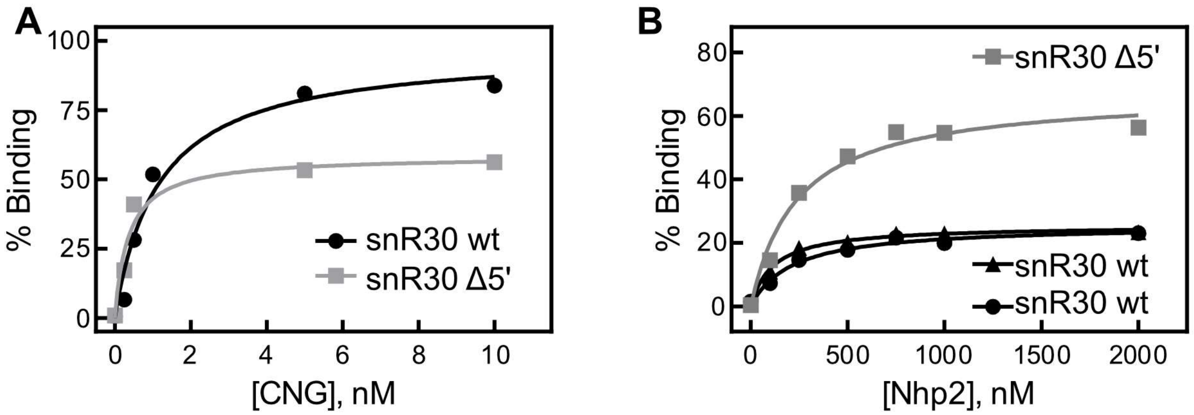

Figure S1. Binding of snR30 by Cbf5-Nop10-Gar1 (CNG) and by Nhp2 alone. A Nitrocellulose filter binding of Cbf5-Nop10-Gar1 binding to full-length snR30 (black circles) and snR30 lacking the 5` hairpin (snR30 Δ5`, grey squares). Hyperbolic fitting of the single replicates yielded dissociation constants of 1.2 ± 0.3 nM and 0.4 ± 0.1 nM for snR30 wt and snR30 Δ5`, respectively. **B** The affinity of Nhp2 alone for snR30 wt (duplicate measurement: black circles and triangles) and snR30 Δ5` (grey squares) was determined by filter binding. The dissociation constants of Nhp2 are 116 ± 17 nM and 212 ± 44 nM for binding to snR30 wt and 240 ± 55 nM for binding snR30 Δ5`, respectively.

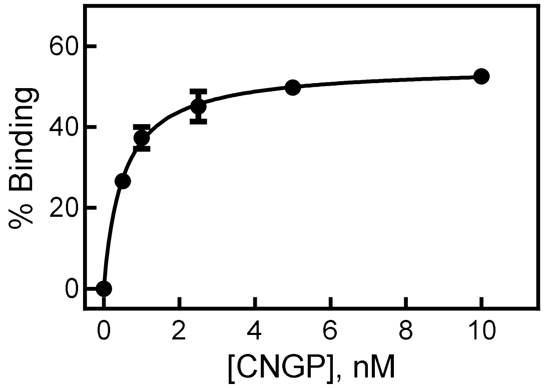

**Figure S2. Binding of Cbf5-Nop10-Gar1-Nhp2 to snR30 lacking the 3` hairpin.** Nitrocellulose filtrations were conducted in triplicate using snR30 Δ3` and increasing concentration of Cbf5-Nop10-Gar1-Nhp2 (CNGP). Hyperbolic fitting of the binding curve determined a dissociation constant of 0.5 ± 0.1 nM.

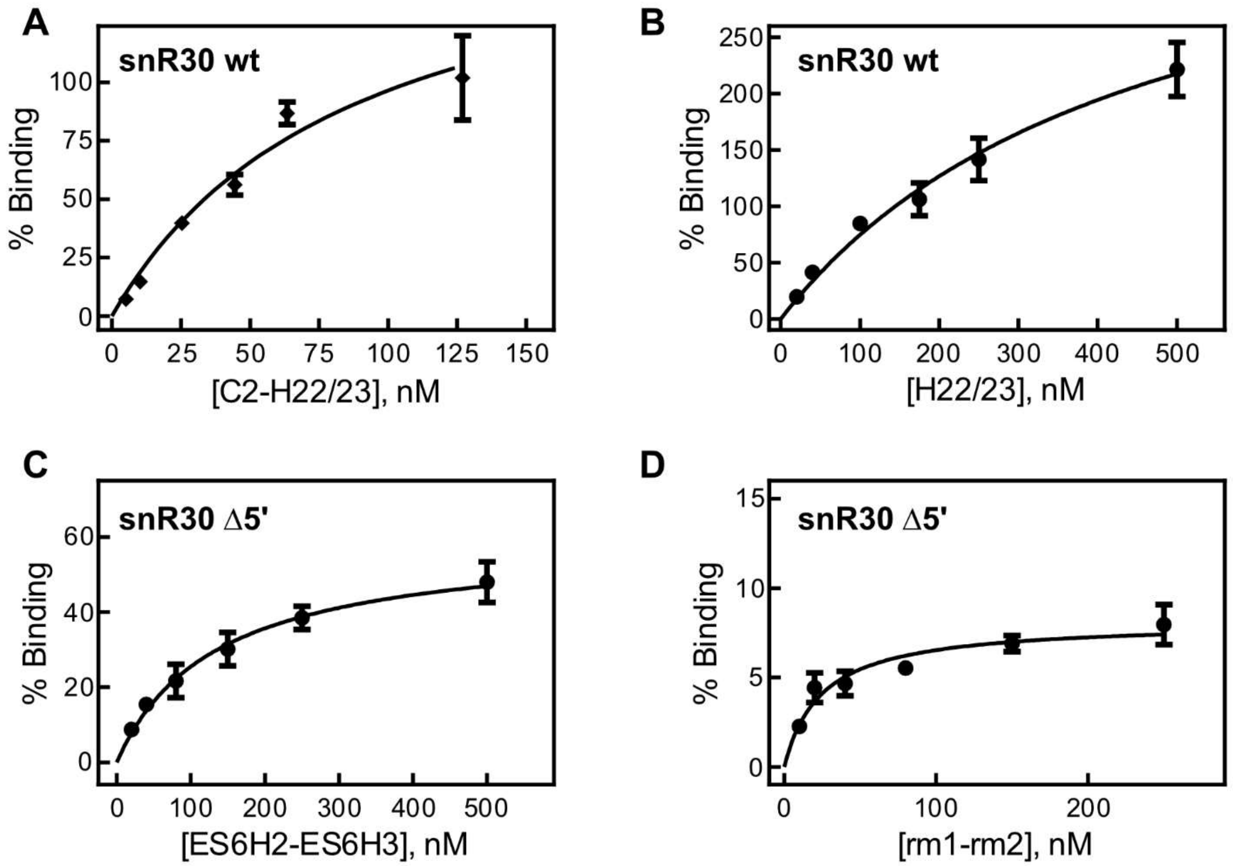

Figure S3. Interaction of the snR30 RNP with rRNA in the presence of competitor RNA. Nitrocellulose filtration assays were conducted using 5 nM of snR30 RNP and increasing concentrations of rRNA fragments in the presence of 1 μM tRNA as competitor. Dissociation constants were determined by hyperbolic fitting and are summarized in Table S1. **A** Binding of the snR30 wt RNP to C2-H22/23. **B** Interaction of the snR30 wt RNP with H22/23. **C** Binding of the snR30 Δ5\` RNP to ES6H2-ES6H3. **D** Interaction of the snR30 Δ5\` RNP with rm1-rm2.

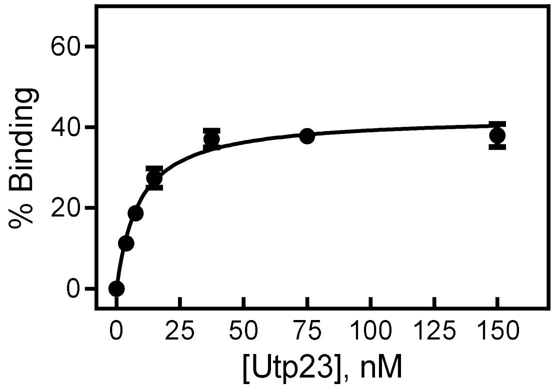

Figure S4. Binding of Utp23 to rRNA in the presence of competitor RNA. Nitrocellulose filter binding was conducted in triplicate titrating Utp23 against the C2-H22/23 fragment of 18S rRNA in the presence of 1 μM tRNA. Hyperbolic fitting determined a dissociation constant of 9.0 ± 1.2 nM

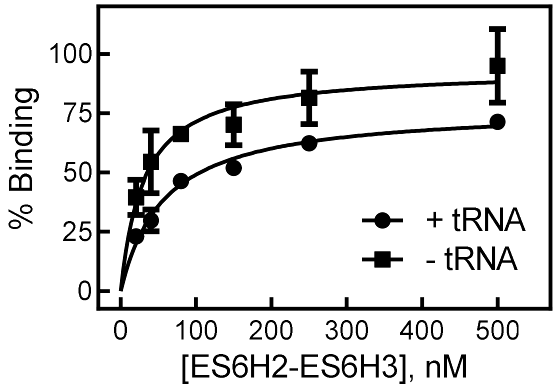

Figure S5. Interaction of the snR30 RNP and Utp23 with rRNA in the presence of competitor RNA. The 18S rRNA fragment comprising two helices of ES6 was titrated against 5 nM of snR30 RNP in the presence of 5 nM Utp23, and rRNA binding was detected by nitrocellulose filtration. The experiment was repeated in triplicate in the absence of competitor RNA (black squares) and in the presence of 1 μM tRNA as competitor (black circles). Hyperbolic fitting yielded dissociation constants of 31 ± 7 nM and 58 ± 8 nM in the absence and presence of competitor tRNA, respectively.
